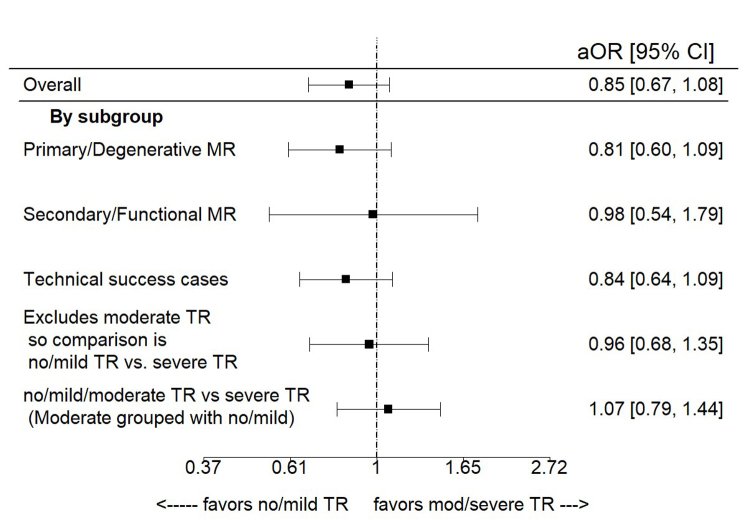# Quality of Life After Mitral Transcatheter Edge-to-Edge Repair According to Baseline Tricuspid Regurgitation

**DOI:** 10.1016/j.shj.2024.100408

**Published:** 2025-01-02

**Authors:** Collin J. Brooks, Neal Duggal, Milan Seth, Megan S. Joseph, Devraj Sukul, Stan J. Chetcuti, Gorav Ailawadi, Himanshu Patel, P. Michael Grossman, Raed Alnajjar, Andrew W. Harris

**Affiliations:** a Department of Internal Medicine, University of Michigan, Ann Arbor, Michigan, USA; b Department of Anesthesiology, University of Michigan, Ann Arbor, Michigan, USA; c BMC2 Cardiovascular Consortium, Ann Arbor, Michigan, USA; d Division of Cardiovascular Medicine, Department of Medicine, University of Michigan, Ann Arbor, Michigan, USA; e Department of Cardiac Surgery, University of Michigan, Ann Arbor, Michigan, USA; f Division of Cardiothoracic Surgery, Henry Ford Health, Clinton Township, Michigan, USA

**Keywords:** Health-related quality of life (QoL), Mitral transcatheter edge-to-edge repair (M-TEER), Mitral regurgitation (MR), Tricuspid regurgitation (TR), Tricuspid transcatheter edge-to-edge repair (T-TEER)

## Abstract

**Background:**

There is a high prevalence of significant tricuspid regurgitation (TR) in patients undergoing mitral transcatheter edge-to-edge repair (M-TEER). Significant TR is associated with poor prognosis and affects decision-making between M-TEER and concomitant mitral and tricuspid valve surgery. Improved quality of life (QoL) is an important metric for patients.

**Methods:**

We analyzed data from 1838 patients undergoing M-TEER included in a multicenter statewide registry from 2015 to 2023. QoL was assessed using baseline and 30-day Kansas City Cardiomyopathy Questionnaire (KCCQ) scores. Patients were classified as no/mild TR or moderate/severe TR, and changes in KCCQ scores were compared. The primary outcome was an adjusted analysis consisting of survival to 30 days with a ≥15-point improvement in KCCQ score.

**Results:**

Complete endpoint data were available for 1421 patients (77.3%). On average, patients undergoing M-TEER experienced large improvements in QoL, regardless of baseline TR group. Thirty-day mortality for patients with moderate/severe TR was higher than for those with no/mild TR (42 [4.1%] vs. 16 [2.0%], respectively, *p* ​= ​0.018). The majority of patients survived to 30 days with ΔKCCQ ≥15 (63.8% for no/mild TR vs. 59.6% for moderate/severe TR, *p* ​= ​0.120). Patients with moderate/severe TR exhibited no difference in the primary adjusted outcome (adjusted odds ratio: 0.851, *p* ​= ​0.177).

**Conclusions:**

The majority of patients experience a large improvement in QoL after M-TEER, regardless of baseline TR severity. Further research should explore a staged approach of M-TEER followed by tricuspid valve intervention as needed compared to concomitant mitral/tricuspid valve surgery.

## Highlights

• Tricuspid regurgitation (TR) is associated with adverse outcomes after mitral transcatheter edge-to-edge repair (M-TEER).
• Data are limited regarding the effect of TR on quality of life (QoL) after M-TEER.
• Most patients with moderate/severe TR had large improvements in QoL after M-TEER.
• QoL outcomes were similar for patients with moderate/severe TR compared to no/mild TR.
• Future studies should compare treatment strategies for patients with concomitant mitral regurgitation and TR.

## Graphical abstract

Left: Unadjusted changes in KCCQ score from baseline to 30 days after M-TEER according to baseline TR group. Right: Comparison of adjusted primary outcome of survival to 30 days and ΔKCCQ ≥15 and adjusted analysis according to subgroups. Abbreviations: KCCQ, Kansas City Cardiomyopathy Questionnaire; TR, tricuspid regurgitation; M-TEER, mitral transcatheter edge-to-edge repair; MR, mitral regurgitation.

**Figure:**
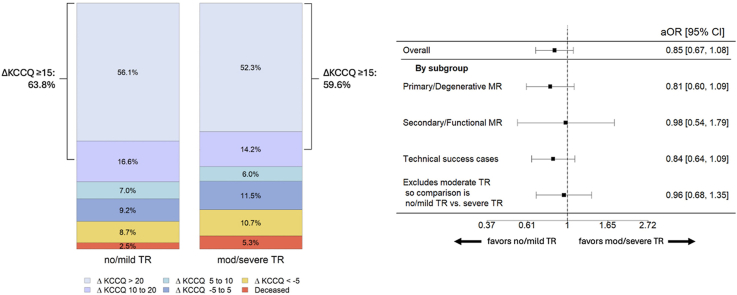

## Introduction

Significant tricuspid regurgitation (TR) is frequently present in patients undergoing mitral valve interventions.^1^, ^2^, ^3^ Decision-making between surgery and transcatheter treatment of mitral regurgitation (MR) is often more challenging in the context of significant concomitant TR. Patients being considered for mitral transcatheter edge-to-edge repair (M-TEER) are often older and have more comorbid conditions, including frailty, than those undergoing surgery for MR.^4^ Although surgery can provide a definitive treatment for both valve lesions, M-TEER has evolved to become a highly successful procedure for the management of MR in appropriately selected patients.^5^, ^6^, ^7^, ^8^, ^9^ However, patients undergoing M-TEER may require additional procedures for staged treatment of TR.

Multiple prior studies have demonstrated that significant TR is generally associated with a poor prognosis and with increased symptomatic burden.^10^^,^^11^ Patients with significant TR undergoing M-TEER have higher rates of mortality than those without significant TR undergoing intervention.^12^, ^13^, ^14^, ^15^ However, patients with more significant TR also had higher rates of atrial fibrillation, chronic kidney disease, anemia, and increased pulmonary artery pressure, which likely also significantly contribute to differences in survival.^12^ In the absence of supportive data from randomized trials, it remains challenging to definitively establish that TR itself is responsible for these differences in survival rather than being a marker of a more significant underlying heart disease.^16^^,^^17^ Up to this point, randomized trials evaluating treatment of TR have failed to show a benefit in hard endpoints of TR intervention. Gammie et al. randomized patients with severe degenerative MR and those at risk for significant postoperative TR (moderate TR, tricuspid annulus diameter >21 mm/m^2^) to mitral valve surgery alone vs. mitral valve surgery plus concomitant tricuspid valve annuloplasty repair.^18^ Although patients undergoing tricuspid annuloplasty did show a decrease in postoperative TR, there were no differences in mortality or heart failure hospitalizations during follow-up. Likewise, the TRILUMINATE trial randomized patients with symptomatic severe TR to tricuspid TEER (T-TEER) and optimal medical therapy vs. optimal medical therapy alone.^19^ There were no differences in mortality or heart failure hospitalizations between the 2 groups. Patients randomized to T-TEER did demonstrate significant improvements in quality of life (QoL), with improvements proportional to the degree of TR reduction.

In the absence of data indicating improvements in hard endpoints in patients undergoing tricuspid valve intervention to date, there is a need to better understand changes in health-related QoL after M-TEER in patients with significant TR. QoL is an important metric for patients and helps to inform shared decision-making. Arnold et al.^9^ previously demonstrated significant improvements in QoL in patients undergoing M-TEER with an average improvement in Kansas City Cardiomyopathy Questionnaire (KCCQ) overall summary score of 29.1, consistent with large improvements in QoL. In this study, improvements in QoL were present at 30 days and remained stable at 1-year follow-up. Prior studies evaluating the effect of baseline TR on QoL outcomes after M-TEER procedures are sparse. We hypothesized that patients with significant TR (moderate/severe TR) at baseline would experience less improvement in QoL following M-TEER procedures than patients with no/mild TR.

## Materials and Methods

### Population

We included patients ≥18 years of age who underwent commercial M-TEER for MR during the period of January 2015 to June 2023 utilizing data collected by the Michigan Structural Heart Consortium (MISHC). MISHC is a consortium that includes 30 hospitals throughout Michigan. Data from Transcatheter Valve Therapy (TVT) Registry forms were available for patients undergoing M-TEER at participating hospitals during this time period. Baseline and 30-day follow-up collection forms were the primary sources of data; 1-year follow-up was utilized when available. Analysis involved consecutive patients with baseline KCCQ data available undergoing M-TEER, including both primary and secondary MR. Outcomes were compared for 2 groups according to baseline TR severity: 1) no/mild TR (which includes no TR, trace TR, and mild TR) and 2) moderate/severe TR.

### Study Outcomes

The assessment of health-related QoL utilizing the KCCQ has been well validated in patients with symptomatic valve disease, including MR and TR.^9^^,^^20^, ^21^, ^22^ Patient QoL was assessed at preprocedure (baseline) and 30-day time points using the KCCQ-12 summary score. One-year change in KCCQ scores was assessed when available. The primary outcome was a composite outcome consisting of 1) survival to 30 days and 2) improvement from baseline to 30-day KCCQ score of ≥15 points, according to baseline TR group and adjusted for relevant covariates. The threshold of 15 points was chosen to be consistent with the primary outcome of the recently reported TRILUMINATE trial.^19^

Secondary outcomes consisted of mean change in KCCQ score from baseline to 30 days and baseline to 1 ​year, as well as survival to 30 days and 1 ​year. Due to significant differences between primary and secondary MR, we also conducted the above analyses after stratifying patients into primary or secondary MR groups. The following key sensitivity analyses were also performed: 1) including only patients who underwent M-TEER with expected device performance (defined by postprocedure transthoracic echocardiogram [TTE] with all of the following: moderate or less MR, at least 1 grade reduction in MR, and mean gradient <6 mmHg) and 2) comparing outcomes for only patients with severe TR (excluding moderate TR) to those with no/mild TR.^23^

### Statistical Analysis

Continuous variables were compared between groups using Student *t*-tests where normality could reasonably be assumed; if not, then Wilcoxon rank sum tests were used. Categorical variables were compared between groups using Pearson chi-square tests. Estimated mortality at 1 ​year by group and associated standard errors were assessed using Kaplan-Meier analysis using the R package “survival.”^24^^,^^25^ The Cox proportional hazards model was utilized to compare 1-year mortality risk according to baseline TR. In the primary endpoint analysis, confounding by baseline covariates and the potential impact of nonrandom patterns of follow-up resulting in missingness were accounted for by utilizing weighted logistic regression models, including inverse probability weighting, so that cases with follow-up data available that had the most similar baseline covariates to cases without follow-up data were given more weight such that they might “stand in” for cases where endpoint data were not available. Baseline covariates used in the multivariable model are listed in Supplemental Table 1, which represent a large number of clinical characteristics available within the TVT registry with potential association with clinical outcomes. These variables are inclusive of variables used by Sammour et al.^15^ in a similar analysis, with additional variables included to limit unmeasured confounding variables between baseline TR groups. Missing baseline covariate values were imputed prior to estimating weights and fitting outcome models using the classification and regression tree method implemented in the R package mice.^26^^,^^27^

## Results

### Baseline Characteristics

A total of 1838 patients who underwent M-TEER in Michigan from 2015 to 2023 met inclusion criteria. Of these patients, 802 (43.6%) exhibited no/mild TR, while 1036 (56.4%) had moderate/severe TR at baseline. The majority of patients, 1428 (78.2%), had primary MR—614 (43.0%) were associated with no/mild TR compared to 814 (57.0%) with moderate/severe TR (*p* ​= ​0.340) (Table 1). Secondary MR was reported in 585 (32.1%) cases, and 208 (11.4%) patients were reported as having mixed MR etiology.

**Table 1:** Baseline characteristics

	All patients (N ​= ​1838)	No/mild TR (n ​= ​802)	Moderate/severe TR (n ​= ​1036)	*p* value
Age (y)	77.2 ​± ​10.3	75.8 ​± 10.3	78.4 ​± ​10.1	<0.001
Male sex	1015 (55.2)	484 (60.3)	531 (51.3)	<0.001
STS predicted risk of operative mortality for mitral valve repair (%)	6.41 ​± ​5.84	5.26 ​± ​4.73	7.31 ​± ​6.44	<0.001
Race				
White	1597 (86.9)	709 (88.4)	888 (86.0)	0.040
Black/African American	207 (11.3)	78 (9.7)	129 (12.5)	0.087
Asian	12 (0.7)	3 (0.4)	9 (0.9)	0.316
American Indian/Alaskan Native	4 (0.2)	2 (0.3)	2 (0.2)	1.000
Native Hawaiian/Pacific Islander	4 (0.2)	2 (0.3)	2 (0.2)	1.000
Health history				
Prior myocardial infarction	586 (31.9)	272 (33.9)	314 (30.3)	0.111
Heart failure	730 (39.7)	300 (37.4)	430 (41.5)	0.083
Ischemic cardiomyopathy	426 (23.2)	181 (22.6)	245 (23.6)	0.625
Carotid artery disease	220 (15.3)	106 (16.4)	114 (14.5)	0.359
Peripheral arterial disease	376 (20.5)	167 (20.8)	209 (20.2)	0.777
Atrial fibrillation/flutter	1229 (66.9)	445 (55.5)	784 (75.7)	<0.001
Hypertension	1677 (91.2)	714 (89.0)	963 (93.0)	0.004
Diabetes	587 (31.9)	255 (31.8)	332 (32.0)	0.949
Current tobacco use	148 (8.1)	82 (10.2)	66 (6.4)	0.003
Prior CVA	228 (12.4)	93 (11.6)	135 (13.0)	0.393
Prior TIA	167 (9.1)	64 (8.0)	103 (9.9)	0.171
Chronic lung disease	751 (40.9)	335 (41.8)	416 (40.2)	0.515
Home oxygen	277 (15.1)	113 (14.1)	164 (15.8)	0.333
Liver disease	49 (2.7)	20 (2.5)	29 (2.8)	0.797
Porcelain aorta	9 (0.5)	7 (0.9)	2 (0.2)	0.083
Hostile chest	71 (3.9)	33 (4.1)	38 (3.7)	0.711
Aortic stenosis	232 (12.6)	85 (10.6)	147 (14.2)	0.026
Dialysis-dependent	76 (4.1)	26 (3.2)	50 (4.8)	0.116
Serum creatinine (mg/dL)	1.50 ​± ​1.15	1.40 ​± ​1.00	1.57 ​± ​1.25	0.002
Hemoglobin level (g/dL)	12.08 ​± ​2.01	12.28 ​± ​1.94	11.93 ​± ​2.05	<0.001
Heart failure hospitalization (<1 y)	890 (48.4)	340 (42.4)	550 (53.1)	<0.001
NYHA class				<0.001
I	14 (0.8)	6 (0.8)	8 (0.8)	
II	304 (16.6)	158 (19.8)	146 (14.1)	
III	1161 (63.4)	508 (63.7)	653 (63.2)	
IV	351 (19.2)	125 (15.7)	226 (21.9)	
Left ventricular ejection fraction (%)	47.5 ​± ​15.5	47.9 ​± ​15.4	47.2 ​± ​15.6	0.326
Left ventricular internal systolic dimension (cm)	4.1 ​± ​1.2	4.2 ​± ​1.2	4.0 ​± ​1.2	0.085
Left ventricular internal diastolic dimension (cm)	5.4 ​± ​1.0	5.5 ​± ​1.0	5.4 ​± ​0.9	0.008
MR etiology				
Primary	1428 (78.2)	614 (77.1)	814 (79.1)	0.340
Secondary	585 (32.1)	250 (31.4)	335 (32.6)	0.638
Mixed	208 (11.4)	76 (9.5)	132 (12.8)	0.035
Procedure history				
Implantable cardioverter defibrillator	360 (19.6)	152 (19.0)	208 (20.1)	0.587
Cardiac resynchronization therapy defibrillator	175 (9.5)	83 (10.3)	92 (8.9)	0.325
Permanent pacemaker	448 (24.4)	169 (21.1)	279 (26.9)	0.004
Prior PCI	642 (34.9)	308 (38.4)	334 (32.2)	0.007
Coronary artery bypass graft	472 (25.7)	196 (24.4)	276 (26.6)	0.309
Surgical aortic valve replacement	120 (6.5)	51 (6.4)	69 (6.7)	0.870
Transcatheter aortic valve replacement	112 (6.1)	56 (7.0)	56 (5.4)	0.192
Tricuspid valve procedure	21 (1.1)	10 (1.2)	11 (1.1)	0.882
Pulmonic valve procedure	3 (0.2)	1 (0.1)	2 (0.2)	1.000

*Notes*. Values are n (%) or mean ​± ​SD.

Abbreviations: CVA, ​cerebrovascular accident; MR, mitral regurgitation; NYHA, New York Heart Association; PCI, ​percutaneous coronary intervention; STS, ​Society of Thoracic Surgeons; TR, ​tricuspid regurgitation; TIA, ​transient ischemic attack.

Patients with moderate/severe TR tended to be older and had a higher prevalence of aortic stenosis, atrial fibrillation/flutter, pacemaker placement, current tobacco use, hypertension, lower hemoglobin, higher serum creatinine, more recent heart failure hospitalizations, and higher surgical risk scores (Table 1). There were no significant differences between the groups regarding left ventricular ejection fraction or the prevalence of prior aortic valve procedures, coronary artery bypass grafting, stroke, diabetes, or myocardial infarction.

### Procedural Outcomes and Mortality

Fifty-eight (3.2%) patients died within 30 days of M-TEER (Table 2). At 30-day follow-up, 417 (22.7%) patients were alive but lacked KCCQ follow-up data. In total, 1421 (77.3%) patients had complete endpoint data, without significant differences in available endpoint data between baseline TR groups.

**Table 2:** Procedural outcomes

	Overall (n ​= ​1838)	No/mild TR (n ​= ​802)	Moderate/severe TR (n ​= ​1036)	*p* value
30-d mortality	58 (3.2)	16 (2.0)	42 (4.1)	0.018
1-y cumulative mortality estimate (95% CI)	17.8% (15.8%-19.8%)	13.7% (10.9%-16.5%)	21.0% (18.0%-23.8%)	<0.001
30-d KCCQ data available	1363 (72.4)	617 (76.9)	746 (72.0)	0.018
Baseline KCCQ	43.0 ​± ​23.5	45.8 ​± ​23.7	40.9 ​± ​23.2	<0.001
30-d KCCQ∗	68.9 ​± ​24.5	71.9 ​± ​23.6	66.5 ​± ​25.0	<0.001
30-d ΔKCCQ∗	24.7 ​± ​25.3	25.4 ​± ​24.4	24.2 ​± ​26.1	0.384
Complete endpoint data available	1421 (77.3)	633 (78.9)	788 (76.1)	0.162
Primary endpoint achieved (30-d survival ​+ ​ΔKCCQ >15)†	874 (61.5)	404 (63.8)	470 (59.6)	0.120
One-year KCCQ data available	768 (41.8)	360 (44.9)	408 (39.4)	0.020
One-year KCCQ∗	72.5 ​± ​23.7	76.0 ​± ​22.6	69.5 ​± ​24.2	<0.001
One-year ΔKCCQ∗	26.3 ​± 26.4	27.3 ​± ​25.9	25.4 ​± ​26.9	0.318
Follow-up echocardiogram results				
Follow-up echo available	1783 (97.0)	778 (97.0)	1005 (97.0)	0.084
Technical success‡	1389 (82.6)	614 (82.9)	775 (82.4)	0.838
MR grade not reported	55	24	31	
No MR	39 (2.2)	21 (2.7)	18 (1.8)	<0.001
Trace MR	226 (12.7)	118 (15.2)	108 (10.7)	
Mild MR	930 (52.2)	409 (52.6)	521 (51.8)	
Moderate MR	427 (23.9)	176 (22.6)	251 (25.0)	
Moderate-to-severe MR	77 (4.3)	19 (2.4)	58 (5.8)	
Severe MR	84 (4.7)	35 (4.5)	49 (4.9)	
Mean mitral valve gradient (mmHg)§	4.4 ​± ​2.5	4.4 ​± ​2.5	4.4 ​± ​2.5	0.811
Postprocedural MV gradient ≥6 mmHg§	201 (10.9)	90 (11.2)	111 (10.7)	0.517
In-hospital outcomes				
In-hospital mortality	24 (1.3)	8 (1.0)	16 (1.5)	0.414
Cardiac arrest	18 (1.0)	6 (0.7)	12 (1.2)	0.518
Stroke	9 (0.5)	4 (0.5)	5 (0.5)	1
Mitral valve reintervention	8 (0.4)	5 (0.6)	3 (0.3)	0.471
Major vascular complication	2 (0.1)	2 (0.2)	0 (0.0)	0.371

*Notes*. Values are n (%) or mean ​± ​SD.

Abbreviations: KCCQ, Kansas City Cardiomyopathy Questionnaire; MR, ​mitral regurgitation; MV, mitral valve; TR, tricuspid regurgitation; TTE, transthoracic echocardiogram.

∗ Only data for those with complete KCCQ follow-up are included.

† Only includes those with complete endpoint data available.

‡ Defined by postprocedure TTE displaying moderate or less MR, at least 1 grade reduction in MR, and mean mitral valve gradient <6 mmHg.

§ Postprocedural MV gradient data were unavailable in 5.3% of cases.

Expected device performance was achieved in the majority of cases and in similar proportions between TR groups (82.9% in no/mild TR vs. 82.4% in moderate/severe TR, *p* ​= ​0.838). Postprocedure TTE displayed improvement to less than moderate MR in 1622 (88.2%) patients overall. In the no/mild TR group, 90.3% of patients displayed moderate or less MR vs. 86.7% for those in the moderate/severe TR group (*p* ​= ​0.007). Elevated postprocedural mitral valve gradients of ≥6 mmHg were seen in 201 patients (10.9%), with a similar proportion reported between TR groups (11.2% in no/mild TR vs. 10.7% in moderate/severe TR, *p* ​= ​0.517) (Table 2). There was no difference in mean mitral valve gradient between TR groups on postprocedural TTE (*p* ​= ​0.811).

**Figure 1:**
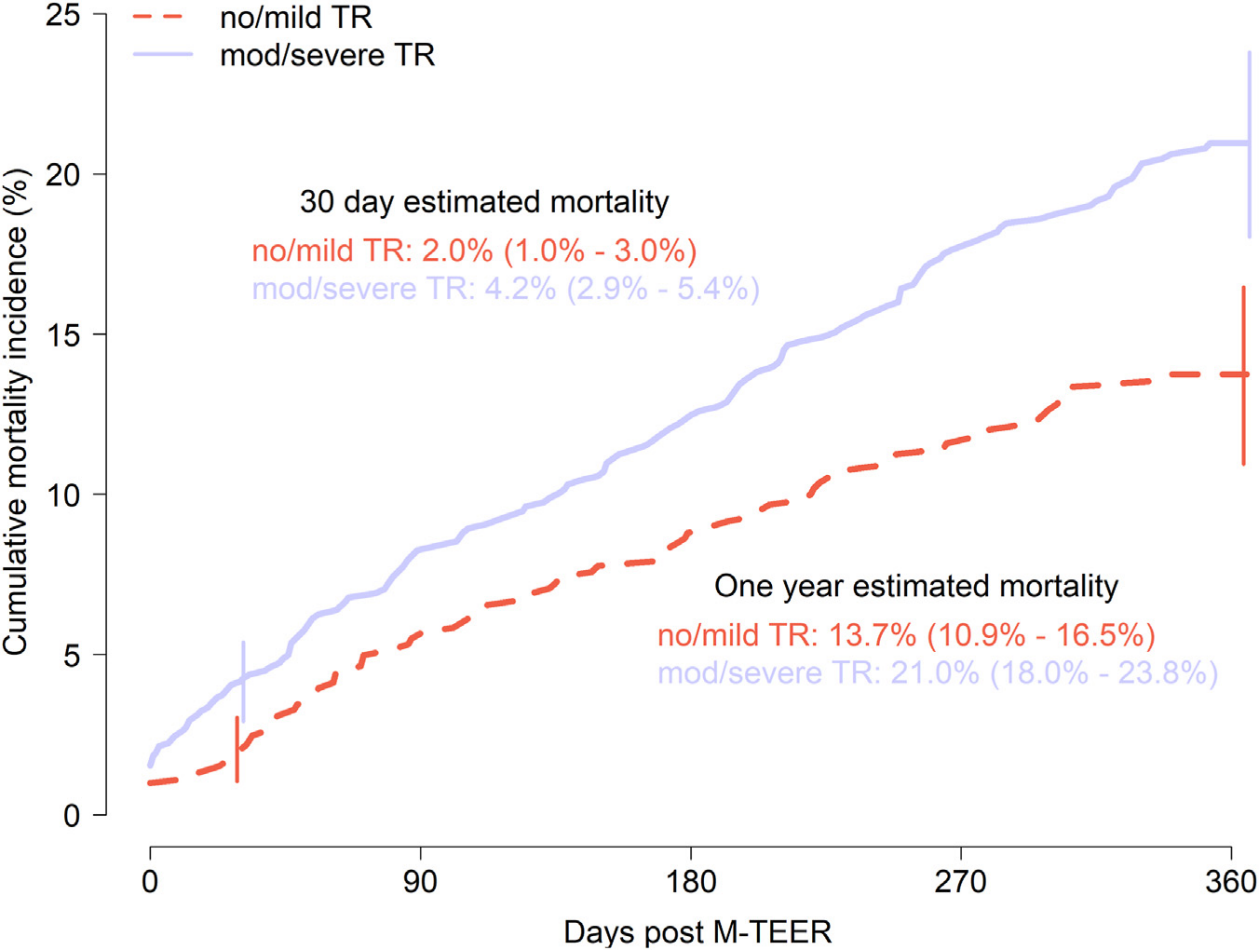
Kaplan-Meier 1-year mortality following M-TEER according to TR severity. Kaplan-Meier curves comparing mortality estimates to 1 ​year for no/mild TR compared to moderate/severe TR groups. Estimated 1-year survival rates with 95% CI are reported, with comparisons made by the log-rank test. Abbreviations: M-TEER, ​mitral tricuspid edge-to-edge repair; TR, ​tricuspid regurgitation.

Procedural mortality was low, with 24 (1.3%) deaths during procedural hospitalization, without significant differences between baseline TR groups. Major complications such as cardiac arrest or stroke were rare, and few patients required reintervention (Table 2). Thirty-day mortality for patients with moderate/severe TR was significantly higher than that of no/mild TR patients (42 [4.1%] vs. 16 [2.0%], respectively, *p* ​= ​0.018) (Table 2). Survival curves separated between TR groups during the first 30 days and continued to diverge through 1 ​year (Figure 1). At 1 ​year postprocedure, moderate/severe TR patients had a significantly higher estimated mortality of 21.0% (18.0%-23.8%) compared to 13.7% (10.9%-16.5%) in the no/mild TR group (*p* ​< ​0.001). A Cox proportional hazards model evaluating 1-year mortality revealed that moderate/severe TR at baseline had an adjusted hazard ratio of 1.785 (95% CI: 1.279-2.491, *p* ​< ​0.001).

### KCCQ Changes

**Figure 2:**
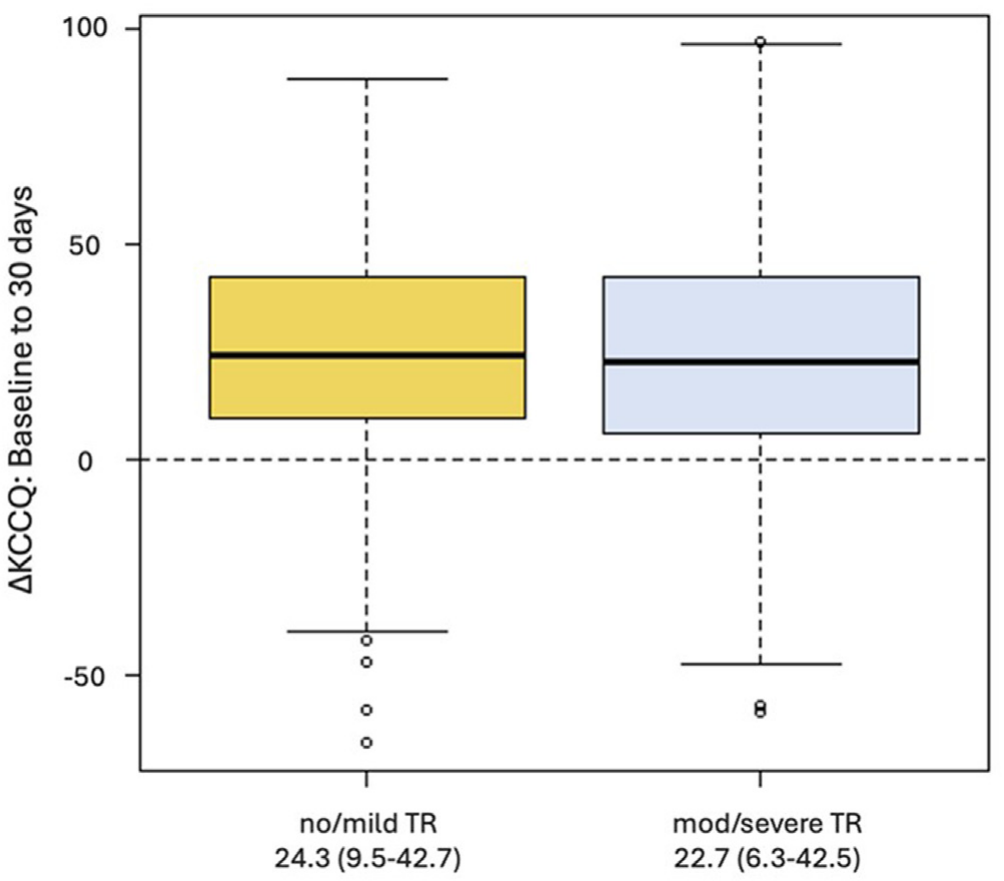
Median 30-day KCCQ change after M-TEER. Box plot displaying change in KCCQ from baseline to 30 days according to TR group. Center line displays median change, boxes represent interquartile range, and whiskers represent 95% CIs. Abbreviations: KCCQ, Kansas City Cardiomyopathy Questionnaire; M-TEER, ​mitral tricuspid edge-to-edge repair; TR, ​tricuspid regurgitation.

**Figure 3:**
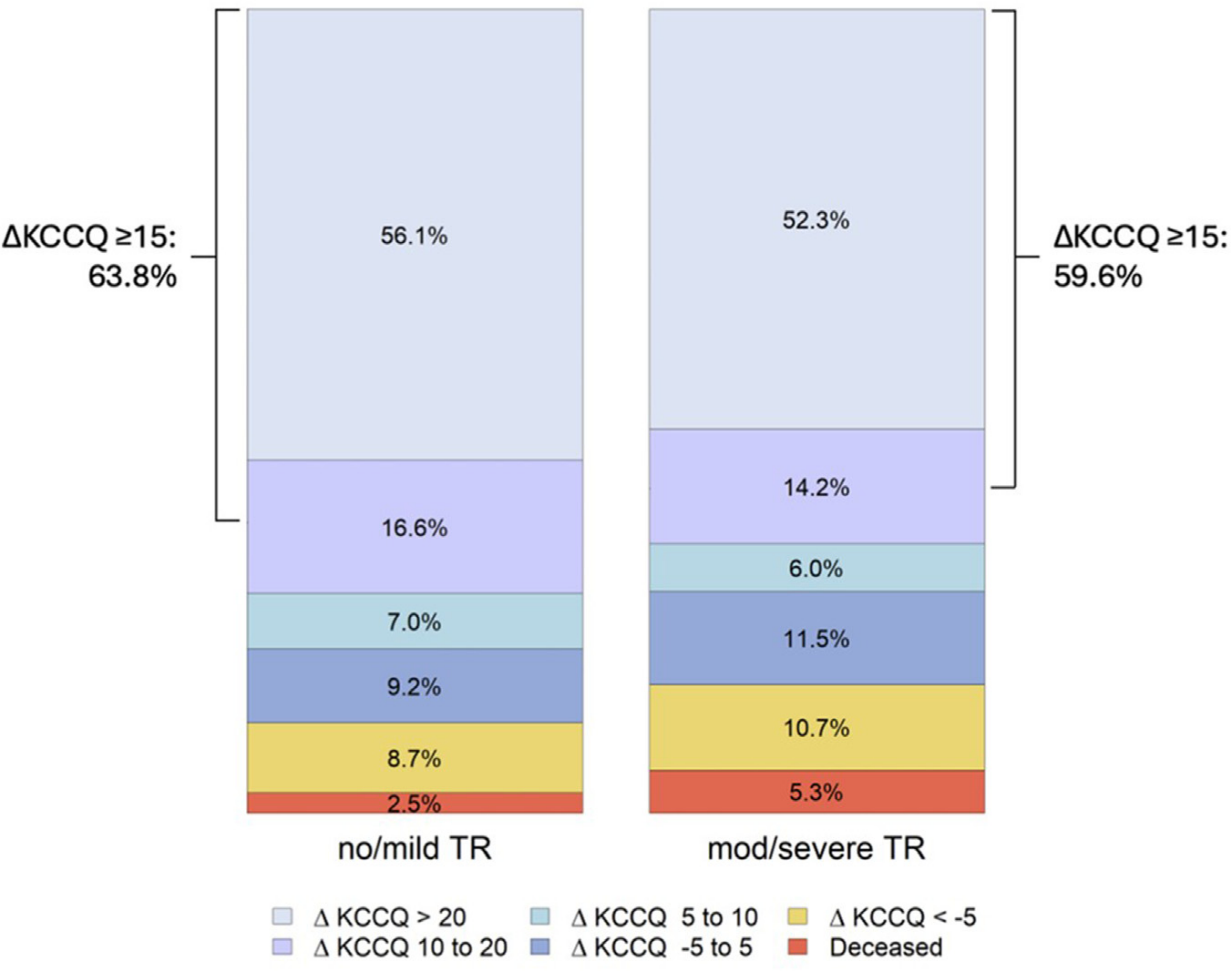
Magnitude of KCCQ change according to baseline TR. Magnitudes of change in baseline to 30-day KCCQ scores according to TR group. Side brackets display the proportion of patients with at least a 15-point improvement. Abbreviations: KCCQ, Kansas City Cardiomyopathy Questionnaire; TR, ​tricuspid regurgitation.

On average, patients demonstrated large improvements in KCCQ scores at 30 days, with similar magnitude of improvement between groups (no/mild TR: 25.4 vs. moderate/severe TR: 24.2, *p* ​= ​0.384) (Figures 2 and 3). Patients with no/mild TR had higher baseline KCCQ scores compared to those with moderate/severe TR (45.8 vs. 40.9, respectively, *p* ​< ​0.001), and this difference remained present 30 days postprocedure (no/mild TR: 71.9 vs. moderate/severe TR: 66.5, *p* ​< ​0.001). Most patients experienced survival to 30 days and a clinically meaningful improvement in KCCQ score of at least 5 points: 79.6% of patients with no/mild TR compared to 72.5% in those with moderate/severe TR (*p* ​= ​0.002). KCCQ data were available in 768 (41.8%) cases 1 ​year postprocedure and continued to display large improvements in QoL. One-year KCCQ scores improved by an average of 26.3 points overall, with no difference in magnitude of change according to baseline TR (no/mild TR: 27.3 vs. moderate/severe: TR 25.4, *p* ​= ​0.318).

### Primary Analysis

**Figure 4:**
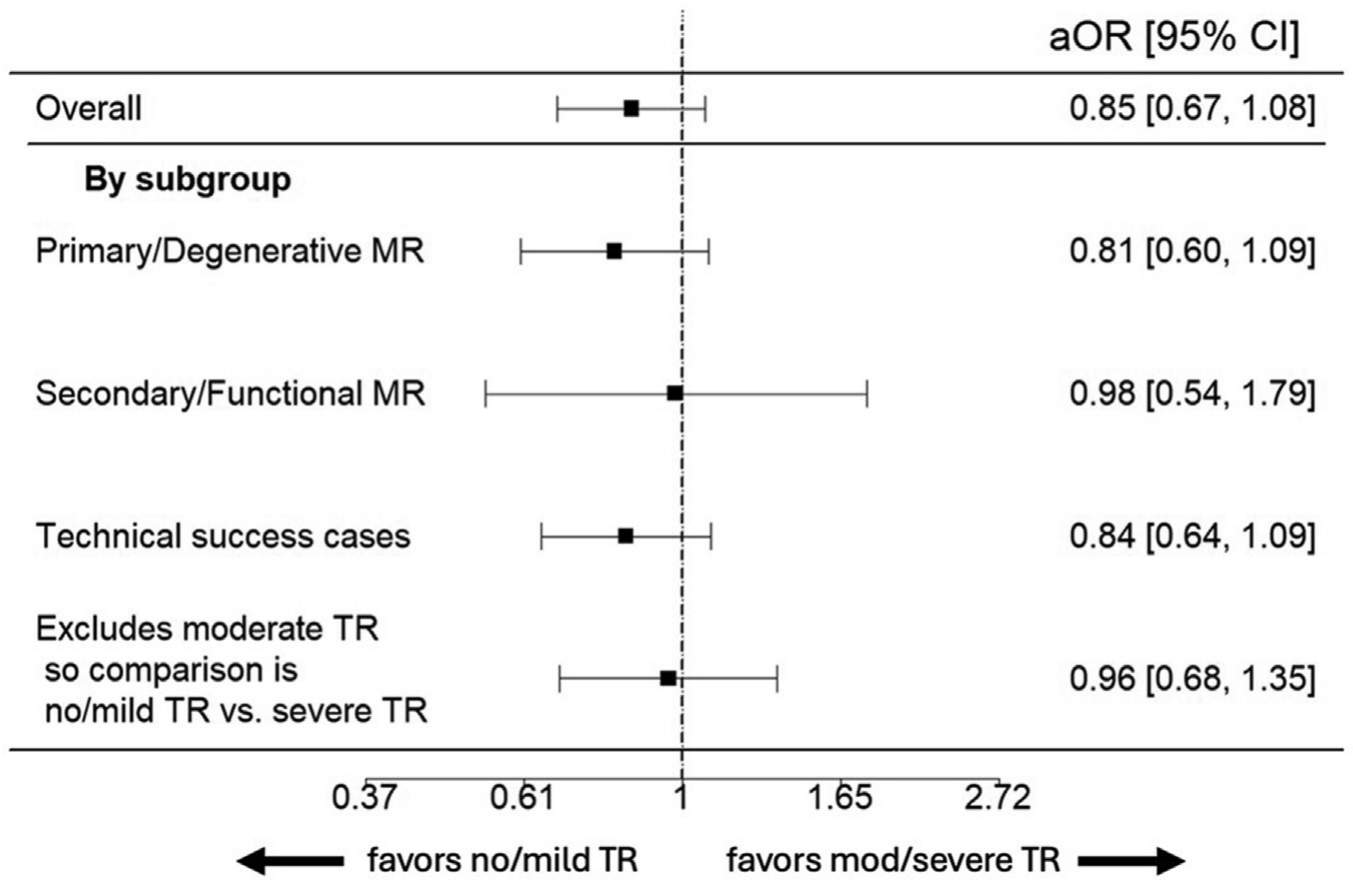
Multivariable-weighted logistic regression: survival with improved quality of life. Forest plot depicting the primary adjusted outcome of survival to 30 days with ≥15-point improvement in baseline to 30-day KCCQ scores. A lower OR favors no/mild TR in likelihood of achieving the primary endpoint. The overall primary adjusted analysis is displayed on the top row, with further subgroup analysis below. Abbreviations: aOR, ​adjusted odds ratio; KCCQ, Kansas City Cardiomyopathy Questionnaire; MR, ​mitral regurgitation; OR, odds ratio; TR, ​tricuspid regurgitation.

Baseline characteristics data were complete. In an unadjusted analysis, a similar proportion of patients in both baseline TR groups achieved the composite endpoint of survival to 30 days with ΔKCCQ ≥15 (no/mild TR: 63.8% vs. moderate/severe TR: 59.6%, *p* ​= ​0.120). In the primary adjusted analysis, patients with moderate/severe TR had a similar likelihood of achieving the composite endpoint of 30-day survival with ΔKCCQ ≥15, with an adjusted odds ratio of 0.851 (95% CI: 0.674-1.076, *p* ​= ​0.177) (Figure 4).

### Secondary Analyses

Multiple secondary analyses were performed to explore the findings from the primary analysis. When stratifying patients according to the etiology of mitral valve disease as primary or secondary, this did not significantly affect our findings. For patients with primary MR, mean changes in KCCQ scores were similar between the baseline TR groups (no/mild TR 24.2 vs. moderate/severe TR 23.1, *p* ​= ​0.491), with similar findings in the adjusted analysis. Likewise, patients with secondary MR demonstrated similar improvements in KCCQ score with mean ΔKCCQ 27.8 for no/mild TR and 26.2 for moderate/severe TR groups (*p* ​= ​0.603). A sensitivity analysis evaluating outcomes in only those patients achieving technical success did not alter the findings of the primary analysis. To ensure that the grouping of patients with moderate TR with patients with severe TR did not bias the results toward the null, we performed a sensitivity analysis comparing only those with severe TR compared to no/mild TR, which also yielded similar results (Figure 4).

## Discussion

In this multicenter observational study of patients undergoing M-TEER, there was no difference in change in QoL according to baseline TR group. On average, patients in both groups experienced large improvements in QoL, with an average improvement in KCCQ score of 25.4 for patients with no/mild TR at baseline vs. 24.2 for patients with moderate/severe TR (*p* ​= ​0.384). In an unadjusted analysis, a similar proportion of patients achieved the primary outcome of survival to 30 days and improvement in KCCQ score of ≥15 (63.8 vs. 59.6% for no/mild TR and moderate/severe TR, respectively, *p* = 0.120). After adjusting for relevant baseline covariates, there remained no significant difference in the primary composite outcome, with an adjusted odds ratio of 0.851 for patients with moderate/severe TR achieving the primary outcome (95% CI: 0.674-1.076, *p* ​= ​0.177). One-year survival was significantly lower for patients with moderate/severe TR at baseline compared to patients without significant TR, which is consistent with prior studies.^12^, ^13^, ^14^

Sammour et al.^15^ recently published a similar analysis of QoL after transcatheter aortic valve replacement and M-TEER procedures according to baseline TR severity utilizing national TVT data. Our findings are complementary to these recently published findings, with some notable differences between our study design. First, though both studies are based on TVT collection forms, MISHC performs additional data auditing, which may improve the accuracy of the reported data compared to national TVT data. In addition, postprocedural MR and mitral valve gradients were not reported in the prior analysis, and mitral valve gradients were not included in the definition of procedural success. In our study, postprocedural mitral valve performance data were nearly complete with postprocedural echocardiographic data available in 97% of patients, resulting in postprocedural MR results available for 97% of patients and mitral valve gradient available for 95% of patients. Postprocedural mitral valve gradients were incorporated into our definition of technical success. Despite this, patients in our study achieved higher rates of technical success without significant differences between baseline TR groups (no/mild TR 82.9% vs. moderate/severe TR 82.4%, *p* ​= ​0.517) compared to data from the national TVT registry (no/mild TR 76.3% vs. moderate/severe 72.1%, *p* ​< ​0.01), which may affect the QoL outcomes reported. The analyses also use different definitions of clinical success, with Sammour et al. utilizing a composite outcome of being “alive and well” (survival, lack of worsening of KCCQ by ​≥10 points, and final KCCQ score of ≥60). Our definition of clinical success was modeled according to the QoL endpoints of the recently reported TRILUMINATE trial as survival with improved KCCQ ≥15. Although both perspectives are reasonable, the concept of “alive and well” biases a successful outcome toward patients with higher baseline KCCQ scores and does not require an improvement in KCCQ score for a successful outcome, which would be the goal of many patients undergoing M-TEER. For example, a patient with a baseline KCCQ score of ≥60 does not need to achieve an improvement in QoL to achieve a successful “alive and well” outcome. We feel that our outcome focusing on survival with significant improvement in QoL provides helpful insight for guiding decision-making with individual patients.

The results of this study are informative for shared decision-making with patients who are weighing options for addressing concomitant mitral and tricuspid valve regurgitation. The results do provide reassurance that even patients with significant baseline TR can expect to have similar improvements in QoL after M-TEER as patients without significant TR. This study replicates findings from numerous studies that patients with significant TR had higher rates of mortality postprocedure than patients without significant TR.^12^, ^13^, ^14^ Although significant TR is associated with worse prognosis even after adjusting for other important conditions such as right ventricular dysfunction and pulmonary hypertension, to date prior randomized studies of tricuspid valve intervention have failed to demonstrate a hard endpoint benefit of tricuspid valve intervention.^19^^,^^28^^,^^29^ Ongoing and future trials evaluating transcatheter treatment options for symptomatic TR will provide further insights into the hard endpoint benefits from tricuspid valve intervention.

In the absence of data on improvement in hard endpoints, as well as evidence of significant improvement in TR severity in approximately one-third of patients after undergoing M-TEER and similar improvement in QoL after M-TEER in the current study, this study supports that a staged approach to managing patients with concomitant MR and TR is a viable option in select patients.^13^^,^^30^ Elderly patients or those with elevated surgical risk could be considered for M-TEER with downstream reevaluation of symptoms and TR severity. Transcatheter tricuspid valve intervention could be considered in patients with persistent severe, symptomatic TR despite mitral valve intervention. One notable limitation to this approach is that not all patients are anatomic candidates for current transcatheter solutions. Evaluation for anatomic suitability for available transcatheter tricuspid valve interventions may be necessary prior to embarking on a nonsurgical approach to treatment of concomitant MR and TR. Further studies evaluating for predictors of persistent TR after M-TEER and changes in tricuspid valve annulus dimensions and other anatomic changes to the tricuspid valve will help to inform patient selection for surgical versus transcatheter treatment approaches.

This study has several notable limitations. First, data are lacking for the severity of TR on follow-up echocardiograms after M-TEER. Data were obtained from the TVT Mitral Clip registry, and follow-up TR severity is not captured within this database. An analysis of the association between changes in QoL and changes in TR severity after M-TEER would provide useful insight but is not available within this dataset. Second, our QoL endpoint data are based on 30-day outcomes rather than 1-year outcomes, which have been frequently utilized in prior studies. Arnold et al.^9^ previously demonstrated similar improvements in QoL at 30 days and 1 ​year for patients undergoing M-TEER. Based on this prior report, we would expect that improvements in QoL shown at 30 days in this study would be durable to at least 1 ​year. This was seen in the subset of patients with 1-year follow-up in our study, though limited by significant missingness at 1 year. Missingness of follow-up KCCQ data is an important consideration in the evaluation of our results. This has been a common limitation in prior studies with similar rates of missingness at 30 days seen in the current analysis compared to prior studies.^9^^,^^31^^,^^32^ Our study population had a higher proportion of patients with primary MR than recently published mitral valve registries, which may be due to Food and Drug Administration approval for the treatment of secondary MR with TEER not occurring until 2019; however, our study had a similar proportion of primary and secondary MR as reported by Sammour et al. utilizing national TVT data (approximately 80% with primary MR).^15^^,^^33^ Finally, the observational nature of this study makes it challenging to interpret the discrepancy between patients with significant TR experiencing similar improvements in QoL after M-TEER and higher mortality rates. Multiple prior studies have demonstrated worse clinical outcomes in patients with significant TR even after adjusting for clinical characteristics; therefore, there may still be residual increased risk of mortality despite improved quality of life. Interestingly, randomized trials of transcatheter tricuspid valve intervention have not demonstrated improvements in mortality despite improved TR and QoL outcomes, suggesting the possibility of residual confounding factors driving differences in mortality in observational studies. Exploring new methodologies for assessing QoL that are specific to symptomatic TR may be helpful in resolving these discrepancies.

## Conclusion

Baseline TR severity did not significantly affect changes in QoL after undergoing M-TEER. The majority of patients experienced a large improvement in QoL regardless of baseline TR severity. With the emergence of transcatheter tricuspid valve interventions, a staged approach to the treatment of concomitant MR and TR with initial M-TEER procedure may be reasonable in select patients. A randomized trial comparing surgical management vs. a staged transcatheter approach should be considered in patients with elevated surgical risk.

## Ethics Statement

The Michigan Structural Heart Consortium (MISHC) consists of 30 participating hospitals throughout the state of Michigan. Participation in the MISHC registry for each hospital has been either approved by or waived by all local institutional review boards as part of continuous clinical care quality improvement initiatives.

## Funding

Support for MISHC is provided by Blue Cross and Blue Shield of Michigan (BCBSM) and Blue Care Network as part of the BCBSM Value Partnerships program. Although BCBSM and MISHC work collaboratively, the opinions, beliefs, and viewpoints expressed by the authors do not necessarily reflect the opinions, beliefs, and viewpoints of BCBSM or any of its employees. Further, BCBSM does not have access to MISHC data, and all patient episodes occurring at engaged hospitals are included in the data registries, regardless of payer.

## Disclosure Statement

R. Alnajjar is a consultant and proctor for Edwards Lifesciences, Abbott, and Intuitive Surgical; is a consultant for Medtronics and Boston Scientific; and is on the advisory board of Ethicon J&J. P. N. Duggal is a consultant for Medtronic. M. Grossman receives research support from Medtronic, Edwards, Abbott, and Gore and is an advisor for Medtronic and Abbott. S. Chetcuti is a consultant for Edwards, Medtronic, Boston Scientific, and PiCardia. H. Patel is a consultant and co-patent holder with W.L. Core & Associates, Inc, and a consultant to Medtronic. G. Ailawadi is a consultant for Medtronic, Abbott, Edwards, Gore, Anteris, Atricure, Philips, Johnson & Johnson, JenaValve, Mediasphere, Arthrex, and Nyra. The other authors had no conflicts to declare.

## Figures and Tables

**Supplemental Figure 1:**
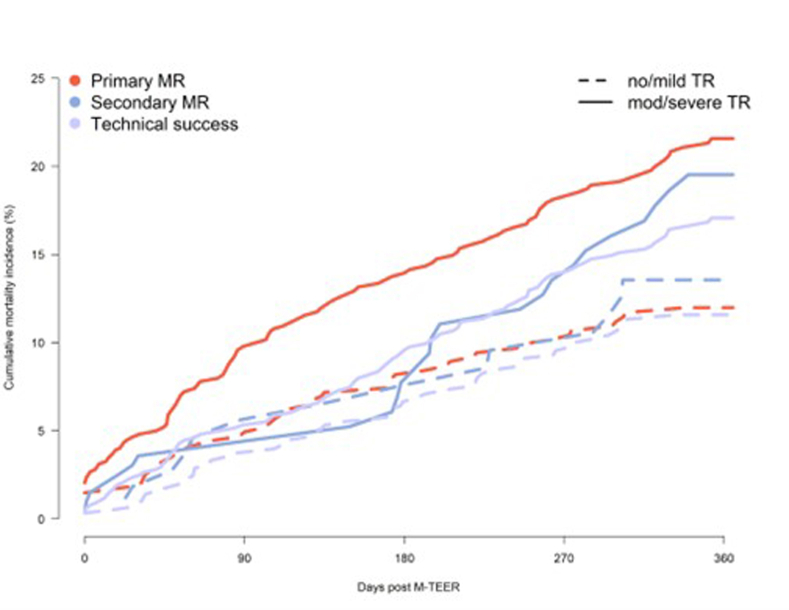


**Supplemental Figure 2:**
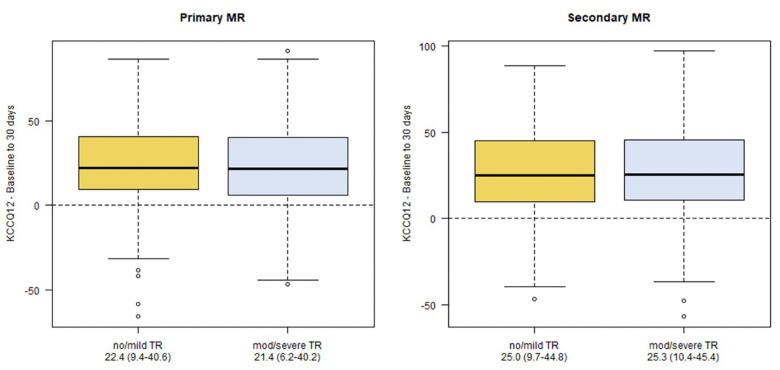


**Supplemental Figure 3a:**
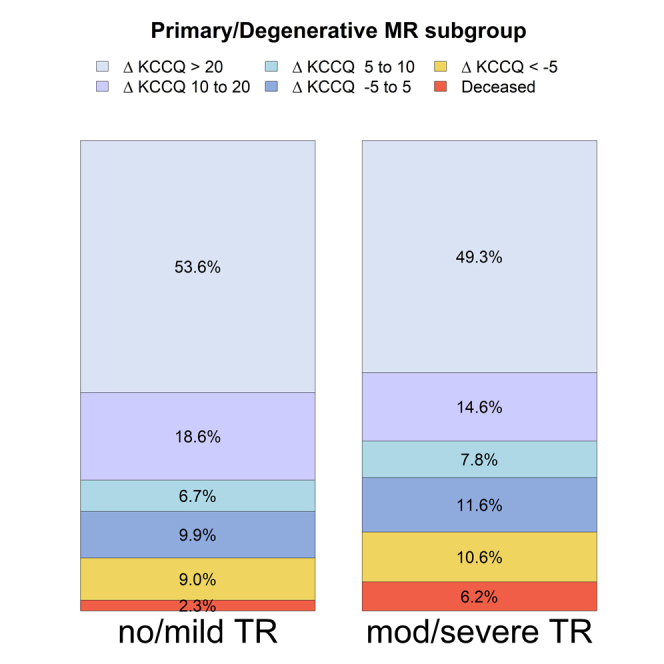


**Supplemental Figure 3a:**
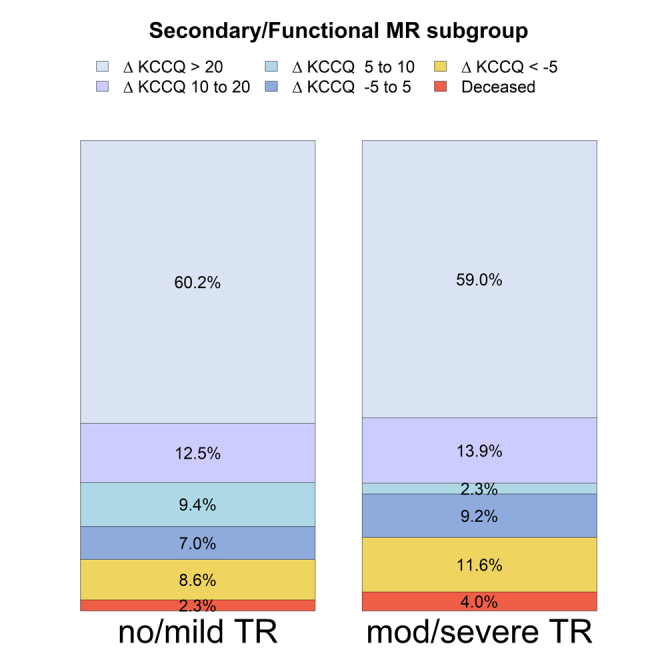


**Supplemental Figure 4:**