# Supplementary material for: Quality of Life After Mitral Transcatheter Edge-to-Edge Repair According to Baseline Tricuspid Regurgitation
Source: Struct Heart. 2025 Jan 2;9(6):100408. doi: 10.1016/j.shj.2024.100408 (PMC12207239; doi:10.1016/j.shj.2024.100408)
Supplement: Supplemental Table 1 [file mmc1.docx]

Supplemental Table 1: Covariates included in primary adjusted analysis

| Age  Sex  STS risk score |
| --- |
| Heart Failure  Cardiomyopathy  Myocardial Infarction  PCI  Coronary Artery Bypass Graft |
| Cerebrovascular Disease  Cerebrovascular Accident/stroke |
| Transient Ischemic Attack (TIA)  Atrial Fibrillation |
| Atrial Flutter  Hypertension  Diabetes Mellitus  Chronic Lung Disease  Home oxygen use |
| Carotid Artery Stenosis  Peripheral Arterial Disease  Pre-procedural serum creatinine  Current dialysis |
| Liver Disease |
| Dementia - Moderate to Severe |
| Aortic Stenosis  Conduction Defect |
| Permanent Pacemaker  Cardiac Resynchronization Therapy  Implantable Cardioverter Defibrillator |
| Prior Aortic Valve Repair Surgery |
| Prior Aortic Valve Replacement Surgery  Prior Aortic Valve Replacement - Transcatheter |
| Prior Aortic Valve Transcatheter Intervention |
| Prior Tricuspid Valve Repair Surgery  Prior Tricuspid Valve Replacement Surgery |
| Prior Other Cardiac Surgery  Prior Aortic Valve Procedure  Prior Aortic Valve Balloon Valvuloplasty  Prior Pulmonic Valve Procedure  Prior Tricuspid Valve Transcatheter Intervention  Prior Mitral Valve Repair Surgery  Porcelain Aorta  Hostile Chest  Endocarditis  COVID-19 Positive |
